# Three-Dimensional Modeling and Quantitative Assessment of Mandibular Volume in Ectodermal Dysplasia: A Case Series

**DOI:** 10.3390/medicina60040528

**Published:** 2024-03-24

**Authors:** Ebru Akleyin, Yasemin Yavuz, Ahmet Yardımeden

**Affiliations:** 1 Department of Pediatric Dentistry, Faculty of Dentistry, Dicle University, Diyarbakır 21010, Turkey; 2 Department of Restorative Dentistry, Faculty of Dentistry, Harran University, Urfa 63000, Turkey; 3 Department of Mechanical Engineering, Faculty of Engineering, Dicle University, Diyarbakır 21010, Turkey

**Keywords:** ectodermal dysplasia, mandible, CBCT, mimics

## Abstract

*Background and Objectives*: Ectodermal dysplasia (ED)—a genetic disorder—is characterized by severe tooth deficiency. We compared the mandibular volume and the sagittal and horizontal mandibular widths between patients with ED (ED group) and individuals without tooth deficiency (control group) using three-dimensional modeling. We hypothesized that the mandibular volume differs in ED cases owing to congenital tooth deficiency. *Materials and Methods*: We used previously obtained cone-beam computed tomography (CBCT) images of 13 patients with ED. The control group data comprised retrospective CBCT images of patients of similar age and sex with a skeletal relationship of class 1. Further, using the three-dimensional image analysis software, the tooth crowns were separated from the mandible, the mandible was reconstructed and the gonion-to-gonion distance in the mandible was marked, the distance to the menton point was measured, and the distance between the two condyles was measured and compared with the control group. *Results*: Overall, 46.2% and 53.8% of the participants were men and women, respectively. In the ED group, the mean age of the participants was 15.46 (range, 6–24) years, and the mean number of mandibular teeth was 4.62. Notably, the edentulous mandible volume of the ED group (27.020 mm^3^) was statistically significantly smaller than that of the control group (49.213 mm^3^) (*p* < 0.001). There was no difference between the two groups in terms of the marked points. For data analysis, the Shapiro–Wilk test, independent samples *t*-test, and Mann–Whitney U test were used. *Conclusions*: It has been considered that mandible volume does not develop in ED cases because of missing teeth. Modern practices, such as the CBCT technique and three-dimensional software, may be effective in identifying the true morphologic features, especially in patients with genetic syndromes affecting the maxillofacial structure.

## 1. Introduction

Ectodermal dysplasia (ED) is a rare genetic disorder characterized by aplasia or dysplasia of ≥2 tissues developing from the ectoderm in the embryo, such as hair, nails, tooth enamel, and skin [1,2]. ED syndrome occurs at a rate of 1 in 10,000 births to 1 in 100,000 births [3]. Clinical characteristics of patients with ED include sparse hair (trichodysplasia), abnormal sweat glands (dyshidrosis), congenital tooth deficiencies (hypodontia or anodontia), abnormal sebaceous glands (asteatosis), abnormal nails (onychodysplasia), protruding lips, sunken midface, saddle-shaped nasal bridge, and an aged facial expression [4,5].

Hypodontia has been known to manifest in 80% of ED cases. In particular, the congenital absence of teeth causes a lack of functional stimulation, leading to atrophy of the alveolar bone [6]. Moreover, reduced growth of the alveolar bone causes excessively narrow and lingually concave alveolar ridges, loss of vertical dimension in the jaws, and developmental retardation in the sagittal direction [6,7].

Owing to missing teeth, such patients need prosthetic rehabilitation at an early age. Notably, implant-supported prostheses can be considered a part of the treatment protocol. However, the congenitally missing teeth and developmental age of children pose difficulties in the placement of oral implants them. In a previous meta-analysis, of 1472 implants in cases with ED, 23.9% of implants were placed in children under 17 years old with ED, and 17.4% of implants failed [8].

Cone-beam computed tomography (CBCT) allows for anatomical and linear measurements of the dental jaw and bone structures [9]. Further, 3D modeling of the oral and maxillofacial structures can be performed using the Mimics software (version 20.0) along with CBCT. Notably, through the use of the segmentation module of the software, a structure can be separated from other neighboring structures, the area and volume values of the segmented structure can be determined, and the densities of the textures can be compared. Accordingly, the implant prostheses to be made for the patient can be planned in advance based on an image exactly matching the patient’s anatomy before the operation. For areas with complex anatomy, the area to be treated can be colored and distinguished from the surrounding tissue [10].

The creation of CBCT-based segmentation models is still a rare procedure in dental practice owing to the generally poor usability of the software and the increased financial costs due to licensing by the manufacturer. However, in cases that require a multidisciplinary treatment approach, such as in patients with ED, the measurement and modeling of mandibular volume, for the first time, may provide insights for further studies.

The aim of this study is to quantitatively measure the mandible volume of ED cases with congenital tooth deficiency by three-dimensional modeling and to compare individuals without tooth deficiency cases. To the best of our knowledge, there is no study similar to the present study in the relevant literature. We hypothesized that the mandibular volume differs in ED cases owing to congenital tooth deficiency.

## 2. Materials and Methods

CBCT images were obtained from 13 Turkish cases of ED and 13 Turkish controls. The subjects were patients of Dicle University Faculty of Dentistry. Scans for the current study were obtained from the available clinical databases and approved by the Institutional Ethics Committee of Dicle University (protocol code 2023-4 and 25 January 2023). Images were acquired using a cone-beam X-ray CT system for dental and maxillofacial use (iCAT 3D Imaging Sciences International, Hatfield, PA, USA) installed at the dental hospital radiology department of our university. No additional CBCT scans were obtained for the current study. CBCT images of patients with ectodermal dysplasia, a very rare disease, were quite limited. CBCT records of the patients were obtained from the archive of the 10-year-old university hospital. It was determined that CBCT imaging was requested for the diagnosis of dental anomalies and excessive tooth deficiency in the ED group, which included pediatric patients, and for orthodontic treatment in the control group. According to the hospital protocol, images are usually acquired in a single 360-degree rotation with a scan time of 8.9 s and a voxel size of 0.4 mm. Further, as a routine image acquisition protocol, the Frankfort plane is set parallel to the horizontal plane, the patients’ heads are oriented, and CBCT is performed while the patients bite at maximum intercuspation. Overall, 13 cases of ED, including 7 women and 6 men (age, 6–24 years), were included in the present study.

Notably, individuals in the control group were of the same age and sex as those in the ED group. Criteria for inclusion in the control group were as follows: individuals with class I skeletal occlusion; those without anterior or posterior crossbite; those without permanent missing teeth (other than third molars); those without severe periodontal bone loss affecting the study area; those with no history of hard tissue surgery (e.g., cyst surgery and resection) in the mandible; those with clear and artifact-free computed tomography images; those presenting a field of view including the entire mandibular bone (condyles, ramus, and body); and those without craniofacial deformities, signs of temporomandibular joint dysfunction, and dental implants.

CBCT images were converted to Digital Imaging and Communications in Medicine (DICOM) format and imported into Mimics 20.0 (Materialise’s Interactive Medical Image Control System; Materialise NV Technologielaan, Leuven, Belgium)—software for three-dimensional (3D) modeling—to obtain 3D reconstructions of the images. In this software, the threshold value can be set based on the scan using the software’s manual segmentation function. Accordingly, volumetric and surface measurements of an object can be made by separating the desired structure from the surrounding structures, and this enables the evaluation of the desired structure using the actual dimensions [11].

A complete manual segmentation of the mandible was performed using the Mimics software. The teeth were separated, and the mandible was reconstructed. After modeling, 3D reconstructive mandible volumes (mm^3^) of the ED and control groups were measured. Further, all landmarks and measurements were determined using Mimics. Notably, menton is the lowest midpoint of the symphysis (in the presence of genital tubercle, the furthest point of the end bone was measured), gonion is the most posterior and lowest point of the mandibular angle, and condylon lateralis is the outermost point of the condyle head [12].

**Figure 1:**
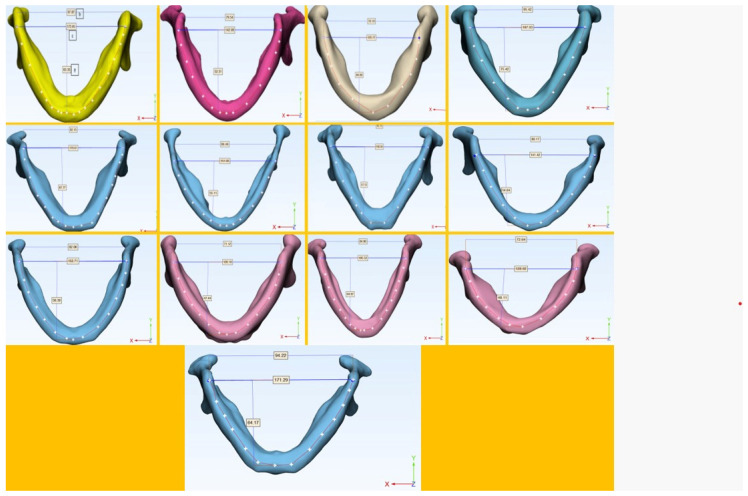
Three-dimensional modeling of the mandible in 13 cases of ectodermal dysplasia. (a) Distance from the gonion-to-gonion distance to the menton point, (b) distance between the most lateral parts of the condyle, and (c) the gonion-to-gonion distance.

In the mandible, the gonion-to-gonion distance was marked, and the distance to the menton point (a), the distance between two condyles (b), and the gonion-to-gonion distance (c) were measured and compared between the ED and control groups (Figure 1). To avoid inter-observer variability, all measurements were performed by an engineer author with experience in using 3D modeling.

### 2.1. Statistical Analysis

The data were analyzed using SPSS (v23, IBM, Armonk, NY, USA). Compliance with the normal distribution was evaluated using the Shapiro–Wilk test. The independent samples *t*-test was used to compare normally distributed variables, whereas the Mann–Whitney U test was used to compare non-normally distributed variables between the two groups. Yates’ correction was used to analyze the categorical variables in both groups. Descriptive statistical values were presented as frequency (percentage), mean ± standard deviation, and median (minimum–maximum). The significance level was set at *p* < 0.050.

### 2.2. Post Hoc Power Analysis

According to the results of the statistical analysis, the power of the study was determined as 99.7% for 26 patients.

## 3. Results

Out of all the participants in both groups, 46.2% were men and 53.8% were women. The mean age of the participants was 15.96 years (Table 1). The mean total number of teeth in the ED group was 11.46, and the mean number of mandibular teeth was 4.62. A statistically significant difference was found between the two groups in terms of the median values of edentulous mandible volume (*p* < 0.001). Notably, the median value of edentulous mandible volume in the ED group was 27.020 mm^3^, whereas it was 49.213 mm^3^ in the control group.

The mean a-point values of the ED and control groups were 58.57 and 63.67 mm, respectively, and there was no statistically significant difference between the two groups in terms of this value (*p* = 0.074). Further, the mean b-point values of the ED and control groups were 85.63 and 87.25 mm, respectively, and there was no statistically significant difference between the two groups in terms of this value (*p* = 0.635). Moreover, the mean c-point values of the ED and control groups were 154.94 and 163.86 mm, respectively, and there was no statistically significant difference between the two groups in terms of this value (*p* = 0.190) (Table 2).

## 4. Discussion

Numerous environmental and genetic factors are known to influence maxillofacial morphologic growth. In particular, hereditary irregularities of the teeth and jaws are among the factors affecting the growth and development of the mandible [13]. This study aimed to evaluate the effect of ED—an inherited syndrome—on the growth of the mandible.

The study by Katayama et al. [14] identified the correlation of mandibular-volume measurement with CBCT and cephalometric values. They concluded that cephalometric radiography is insufficient for a correct understanding of maxillofacial morphology and reported that quantitative measurements obtained from a 3D study of the changes in mandibular volumes to explore the mechanisms controlling mandibular morphogenesis during growth will aid in the diagnosis and treatment selection [14].

Although many studies in the literature have reported various uses of CBCT, those evaluating the volumetric estimation of the mandible are limited [15,16,17]. Moreover, to the best of our knowledge, no study has used 3D modeling for the craniofacial region in patients with Treacher Collins syndrome, except for one study that evaluated the correlation between craniofacial features and nasal airway volume in such patients [18].

Notably, CBCT allows 3D reconstructions of maxillofacial structures [15]. In the present study, all DICOM data were reconstructed using Mimics 20.0 (Materialise NV). Moreover, the scans used in this study were obtained from the same CBCT machine using the same acquisition parameters. Therefore, all factors affecting the accuracy of the 3D modeling were checked before the segmentation process.

Notably, 3D model creation is based on the process of segmentation, which is defined as the virtual separation of irrelevant structures of the specified anatomical region. Manual segmentation has been recognized as the gold standard for 3D rendering maxillofacial structures as it enables the detection of areas with low bone density or areas without well-defined boundaries due to their low contrast and proximity with other structures [11]. In the Mimics software used in the present study, the threshold value was adjusted from the scan using the software’s manual segmentation function. Similarly, Veli et al. [16] and Deguchi et al. [15] separated the mandible from the tooth crown and measured the total volume of the mandible by manual segmentation.

In a study on a group of adult patients with class-1 occlusion, Katayama et al. [14] reported that the mandibular volumes were 63.2 cm^3^ and 55.9 cm^3^ in men and women, respectively. In our study, the mandibular volume of the control group, including class-1 patients, was 49.3 cm^3^. In our study, it was thought that the mandibular volume was lower in the control group due to the age range, which was from 7 to 24.

Tooth agenesis is common in all forms of ED. More et al. [19] reported partial anodontia in 94.74% of 19 ED cases. Further, Akleyin et al. [5] reported missing teeth in all 44 children with ED and indicated that 8 patients had <6 missing teeth. Yavuz et al. reported tooth agenesis, ranging from hypodontia to anodontia, in all 15 patients aged 5–45 years [20]. Similarly, in our study, the total number of teeth in 13 patients with ED was 11.46, and the number of teeth in the mandible was 4.62. In patients with ED, the growth of the jaw is hindered because of severe tooth loss. Moreover, such patients have bimaxillary retrusion relative to the anterior cranial base, decreased vertical dimension, and a skeletal predisposition for class 3, leading to increased jaw protrusion [21,22]. According to Wolff’s law, bones undergo changes in correspondence with the forces applied to them, thereby gaining density and more resistance. Moreover, bones need stimulation to maintain their shape and density [23]. In a long-term study of patients who had lost all their teeth, bone resorption continued throughout the study period, and volume loss was four times greater in the mandible than in the maxilla [24]. According to the results of the present study, we quantitatively found that the skeletal volume of the jaw was almost halved in the ED group compared to the control group due to the small number of teeth in the mandible.

Studies on mandible size in patients with ED have generally focused on two-dimensional analyses based on lateral cephalograms, which assess linear distance and area [2,20,21,25,26]. A multidisciplinary team is required for the functional and aesthetic rehabilitation of patients with ED who have multiple missing teeth and need early treatment in childhood. Hence, it is important to monitor and enhance the growth of jaws to achieve better oral function and facial aesthetics [27]. Data on craniofacial growth associated with ED are scarce and mostly based on case reports [26]. To maximize the outcome of clinical treatments in patients who are still growing and developing, it is important to assess potential ED-specific facial growth patterns using quantitative methods [28]. In a study of 12 patients with ED, in which researchers directly used computational anthropometry, volumetric facial growth patterns were found to demonstrate a slight reduction in overall facial growth compared with controls, with a delay of approximately 2 years in mandibular and maxillary development [28]. Moreover, a cephalometric analysis of a Japanese patient with ED and anodontia between 6 and 18 years of age reported slightly reduced growth of the maxilla but no detectable effect on the mandible [29]. Moreover, Jonhson et al. [25] reported that decreased lower and total facial heights are associated with severe maxillary hypodontia in cases of ED. According to the results of our study, there was no difference between the ED and control groups in terms of the reference point values based on the sagittal and horizontal measurements of the mandible. Thus, it can be concluded that mandibular growth is not affected in cases of ED; further, predisposition for class 3 may be attributed to maxillary hypodontia, but mandibular volume can be reduced due to severe tooth loss. However, more studies are needed to validate these results.

This study has some limitations. When adapting such studies for use in a clinical setting, many factors must be taken into account, such as the appropriate segmentation for model-building, threshold bone pixel values, software algorithm, contrast resolution of the scan, and technical skills of the operator. In the study by Akleyin et al., where the reasons for requesting CBCT in children and adolescents were examined, the rate of requesting CBCT for dental syndromes (0.8%) was remarkably high. This is because the university was located in the southeast of Turkey, where the rate of consanguineous marriages was much higher than in the rest of the country [30]. Although ED images in this study were obtained retrospectively from a 10-year archive, the number of CBCT images and the sample size were small. The mean distance difference from the gonion–gonion distance to the menton point between the ED and control groups was very close to the statistical threshold. In a larger sample group, this may be important. To overcome the limitation of the small sample size, all measurements were performed by the same author, homogenizing the age and sex of the patients. Moreover, the power of this study was found to be high in the power-hoc analysis. Nevertheless, studies with a large sample size are needed for further evaluation.

## 5. Conclusions

Our hypothesis that mandibular volume differs in ED cases due to congenital tooth deficiency was confirmed according to the results of the study. The data obtained in the present study provide important insights to guide future research to understand the effects of ED syndrome on skeletal structure. Based on the results of our study, the use of new modern technologies, such as 3D CBCT and 3D modeling, in clinics and research on genetic syndromes, such as ED, affecting the skeletal structure of the dental jaw can increase the chances of success in the diagnosis and treatment of such cases.

## Figures and Tables

**Table 1:** Comparison of sociodemographic data of the ED and control groups.

	Group		Test Statistics	*p*
	ED	Control		
Sex				
Male	6 (46.2)	6 (46.2)	0.000	1.000 *
Female	7 (53.8)	7 (53.8)		
Age	15.5 ± 5.8	16.5 ± 5.5	−0.45	0.657 **
	16 (6–24)	18 (7–24)		

* Yates’ correction; ** independent samples *t*-test; frequency (percentage); mean ± standard deviation; median (minimum–maximum).

**Table 2:** Comparison of the parameters measured in the ED and control groups.

	Group				*p*
	Patient Group (ED) (*n* = 26)		Control Group (*n* = 26)		
	Mean ± SD Median (Min–Max)		Mean ± SD Median (Min–Max)		
Toothless Mandible Volume (mm^3^)	28,810.62 ± 10,147.95	27,020 (16,618–42,970)	46,852.15 ± 8376.3	49,213 (28,283–58,151)	<0.001 *
Point a (mm)	58.57 ± 8.17	57.12 (47.44–75.42)	63.67 ± 5.5	64.47 (52.52–71.05)	0.074
Point b (mm)	85.63 ± 8.75	84.8 (71.12–97.87)	87.25 ± 8.41	87.17 (67.2–99.07)	0.635 **
Point c (mm)	154.94 ± 19.06	152.71 (126.18–187.03)	163.86 ± 14.35	169.2 (130.86–181.79)	0.190 **

* Mann–Whitney U test; ** independent samples *t*-test; mean ± standard deviation; median (minimum–maximum).

## Data Availability

Data are contained within the article.
